# Role of Dietary Lipids in Modulating Inflammation through the Gut Microbiota

**DOI:** 10.3390/nu11010117

**Published:** 2019-01-08

**Authors:** Paul J. Wisniewski, Robert A. Dowden, Sara C. Campbell

**Affiliations:** Department of Kinesiology and Health and The Rutgers Center for Lipid Research and The Center for Digestive Health, New Jersey Institute for Food, Nutrition and Health, Rutgers University, New Brunswick, NJ 08901, USA; paulw@kines.rutgers.edu (P.J.W.); rad267@rutgers.edu (R.A.D.)

**Keywords:** microbiota, lipids, dybiosis, gut, LPS, obesity, gut permeability

## Abstract

Inflammation and its resolution is a tenuous balance that is under constant contest. Though several regulatory mechanisms are employed to maintain homeostasis, disruptions in the regulation of inflammation can lead to detrimental effects for the host. Of note, the gut and microbial dysbiosis are implicated in the pathology of systemic chronic low-grade inflammation which has been linked to several metabolic diseases. What remains to be described is the extent to which dietary fat and concomitant changes in the gut microbiota contribute to, or arise from, the onset of metabolic disorders. The present review will highlight the role of microorganisms in host energy regulation and several mechanisms that contribute to inflammatory pathways. This review will also discuss the immunomodulatory effects of the endocannabinoid system and its link with the gut microbiota. Finally, a brief discussion arguing for improved taxonomic resolution (at the species and strain level) is needed to deepen our current knowledge of the microbiota and host inflammatory state.

## 1. Introduction 

**Figure 1:**
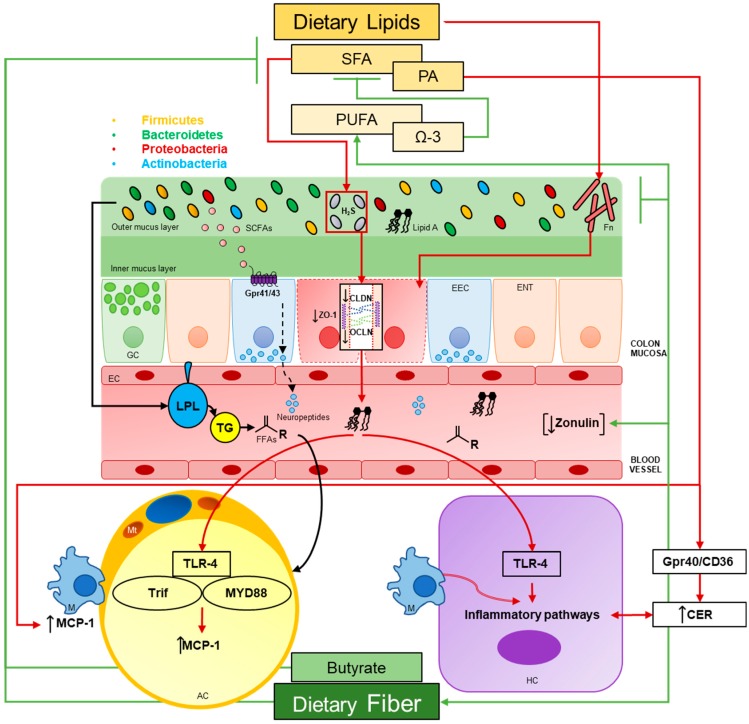
The human gut microbiota is dominated by the main phyla Bacteroidetes, Firmicutes, Actinobacteria and Proteobacteria. Collectively, it contributes to host energy regulation through the production of short-chain fatty acids and subsequent release of neuropeptides from enteroendocrine cells following the activation of short chain fatty acid receptors Gpr41 and 43 (black dotted line arrows), as well as through actions on lipoprotein lipase which stimulates the release of free fatty acids from triglyceride-containing molecules (e.g. chylomicrons) where they are then stored in adipocytes (black arrows). Dietary lipids cause changes in the microbial community structure but lipid type determines whether these ecological shifts prove beneficial or harmful to the host. Saturated fatty acids have recently shown to promote dysbiosis by enriching sulfate reducing bacteria which produce H2S (red arrow, red box). This dysbiosis has shown to degrade epithelial integrity through the suppression of tight junction proteins ZO-1, claudins and occludin as well as to promote gut inflammation (red dotted lines around epithelial cells, dotted lines in insert). Because of this enhanced permeability, passage of LPS into systemic circulation is increased (red arrow, Lipid A molecule). In white adipose tissue, LPS and palmitic acid increase adipocyte inflammation through the upregulation of MCP-1 expression in macrophages and in adipocytes but in a TLR-4-Trif-MYD88 dependent manner (red arrows). In liver, LPS increases hepatocyte inflammation through TLR-4 mediated inflammatory pathways while palmitic acid compounds the activation of such pathways through the activation of free fatty acid receptors Gpr40 and CD36, and a subsequent increase in ceramide de novo synthesis (double-sided red arrow). Like adipocytes, the extent of hepatic inflammation is exacerbated with the presence of macrophages (double-lined red arrow). Interestingly, low serum levels of Zonulin are associated with higher intakes of dietary fiber and polyunsaturated fatty acids (green arrows). Increases in the abundance of Fusobacterium nucleatum, a known risk-factor for colorectal cancer development and a promoter of gut inflammation (red arrow), is associated with high-fat diets (red arrow) but may be reduced in abundance by adequate dietary fiber intake (green T arrow). Lastly, exogenous butyrate, omega-3 fatty acids and dietary fiber have shown to attenuate the deleterious effects of saturated fatty acids on intestinal health (green T arrow). ENT, enterocyte; EEC, enteroendocrine cell; GC, goblet cell; CLDN, claudins; OCLN, occludin; EC, endothelial cell; H2S, dihydrogen sulfide; FFAs, free fatty acids; LPL, lipoprotein lipase; TG, triglyceride; M, macrophage; AC, adipocyte; Mt, mitochondria; HC, hepatocyte; CER, ceramide; Fn, Fusobacterium nucleatum.

The microbiome represents a diverse community of microbes, archaea, and viruses—and their genes—that collectively accounts for a genome 100 times the size of ours. In truth, many scholars claim that host genome and the microbiome, collectively account for a shared “metagenome” [1] often described as our microbial organ [2]. Large international ventures like the National Institute of Health’s Human Microbiome Project (HMP) has enhanced our understanding of these complex communities in and on our bodies [3]. Gut microbial communities have some of the highest cell densities seen in nature, but paradoxically, sparse diversity exists at the super-kingdom level [2]. Bacteria are the predominant members of the gut microbiome, encompassing upwards of 99% of the gene catalogue within the gut [4]. The gut harbors the largest abundances of these microbes (or microbiota), with estimates of 10^13^ bacterial cells [5] and over 1000 different species [4] dominated by four main phyla, Bacteroidetes and Firmicutes (which make up roughly 90–99%), Actinobacteria and Proteobacteria [1]. Importantly, the gut microbiota is highly diverse at the species and strain level. Microbial lineages are inherited from mothers [2] and change over time, particularly during infancy in humans [6,7]. However, the community is stable throughout much of the lifespan and is unique to each individual—approximately 1/3 of the community is similar to parents. In 2010, Qin et al. [4] used metagenomic sequencing to analyze fecal samples from 124 European individuals and found, even with the most common 57 species present in at least 90% of samples, significant interindividual variability occurred, ranging from 12- and 2187-fold differences between communities. However, their results confirmed Bacteroidetes and Firmicutes having the largest abundance of reads [4]. Several species, such as *Bacteroides* spp. create stability in the gut ecosystem with their arsenal of glycoside hydrolases and appropriate polysaccharide-binding proteins, even in the absence of dietary polysaccharides [2]. Arumugam et al. [8] described three robust clusters, or enterotypes within the human gut microbiome, where Bacteriodes, Prevotella and Ruminococcus are represented across 39 samples and cluster divergently from one another. The underlying basis for such enterotype clustering, however, remains unknown [8]. This microbial organ has many functions for the host: harvesting energy from undigested foodstuffs [2]; increased fat storage [9]; synthesizing vitamins [10]; and overall improving colonic health. Recently, rapid alterations of gut microbial communities have been shown to occur as result of dietary alterations [11] (Figure 1). What is important for the host, however, is functional redundancy and community stability remain fairly consistent over time [3] and, thus, key processes remain unaffected by changes in diversity [2]. Therefore, describing an optimal microbiome for host health proves problematic as to the staggering variability from person to person.

For the past few years, there has been considerable interest in the microbiome and its role in health and human disease [12]. Gut dysbiosis occurs when an imbalance between beneficial and harmful bacteria transforms the community, where normally dominating species are outcompeted by otherwise underrepresented microbes increasing in abundance; several groups have shown increases in mucus degradation and subsequent decreases in the intestinal lining, which produces systemic “low-grade inflammation” [13,14,15], which is known to be involved in several chronic diseases, namely obesity and type-2 diabetes [16]. However, what remains to be defined is whether gut dysbiosis is the cause or consequence of host disease state. Still, dysbiosis has been implicated in several host disease states like, obesity [9,14,17] and type-2 diabetes mellitus [2,14,18,19], cancer [20] and inflammatory bowel diseases [21]. An early study out of Jeff Gordon’s lab demonstrated the now widely cited reduction in abundance of Bacteroidetes and proportional increase in Firmicutes in the obese state in mice [2]. In another study, Bakhed et al. [22] examined the effects of the microbiota on host fat storage, showing germ-free (GF) mice (no microbiota) were protected from obesity via upregulation of adenosine monophosphate kinase (AMPK) activity, which elicited increased fatty acid oxidation via acetylCoA carboxylase (ACC-P) and carnitine-palmitoyltransferase (CPT1) in skeletal muscle and liver tissue. Furthermore, knockout mice lacking an inhibitor to lipoprotein lipase, fasting-induced adipose factor (Fiaf), were not protected from dietary-induced obesity, suggesting GF mice are protected from obesity by two independent mechanisms: increased AMPK activity and levels of Fiaf, known to upregulate the peroxisomal proliferator-activated receptor coactivator (PGC-1α) [22]. Turnbaugh et al. showed in 2006 an obese microbiome has the capacity for increased energy harvest where, obese (*Ob/Ob*) ceacal microbiome samples were enriched for environmental gene tags (EGTs) encoding enzymes involved in initial breakdown, further importation and metabolism of indigestible dietary polysaccharides, and also end-products of fermentation, namely short-chain fatty acids (SCFAs) [19]. Obese mice have increase concentrations of butyrate and acetate, probably due to the increase in abundance of Firmicutes phyla; Firmicutes are known butyrate producers [19]. Notably, the effects of *Ob/Ob* microbiome were transferable with fecal transplant to GF mice. Later papers out of Gordon’s lab demonstrated that transplantation of fecal material from obese mice to GF mice, increased fat deposition in the GF mice compared to lean donors [23,24], supporting the contribution of the gut microbiota to obesity. Notably, studies in humans confirm these alterations described in animal models. Vrieze et al. demonstrated the transfer of a lean gut microbiota to obese subjects improved insulin sensitivity in 6 weeks [25]. In a recent study, Zhang et al. demonstrated improvements in patients’ body weight, BMI, fasting glycaemia, lipid profile (total cholesterol, LDL) and systemic markers of inflammation (C-reactive protein and IL6) following a dietary intervention consisting of a diet rich in non-digestible carbohydrates [17]. Of note, [17] showed that similarly, transplantation of a genetically obese human microbiota (Prader-Willi syndrome (PWS)) to GF mice produced larger adipocytes and a higher inflammatory response (increased liver, colonic and ileum TNF-α, TLR4 and IL6 gene expression, respectively) than did GF mice administered a post-intervention transplantation. In 2018, Zhao et al. [26] conducted a clinical trial using the same high fiber diet used by Zhang et al. 2015 [17] to improve hemoglobin A1c (HbA1c) and glucagon-like peptide-1 (GLP-1) production in clinically diagnosed T2DM patients [26]. These studies provide evidence for the transmissible effects of an obese microbiota to impact host physiology; however, the underlying mechanisms remain elusive. In a seminal paper, Cani et al. demonstrated that infusion of lipopolysaccharide (LPS), formed from the degradation of Gram-negative bacteria in the gut, had similar effects when compared to a high-fat diet (HFD) at increasing fasting glycaemia, insulinemia and whole-body, hepatic, and adipose tissue weight gain [14]. Furthermore, the study showed increases in hepatic, but not whole-body insulin resistance, as well as adipose F4/80-postive cells and markers of inflammation were reported with infusion of LPS. Lastly, CD14 mutant mice resisted most of the LPS and HFD-induced features of metabolic disease [14]. Collectively, these studies provide evidence for the role of the gut microbiota to increase host inflammatory responses, and therefore disease status, namely through the production of LPS. In the present review, we address the interaction of the gut microbiota in modulating host inflammatory responses in the context of dietary lipid consumption. Particular attention is paid to the role of microorganisms in host energy regulation, LPS and HFD-induced inflammation, as well as associated changes in the gut microbiota leading to increased gut permeability. We also highlight exciting new areas of microbiome research regarding dietary protein intake and the endocannabinoid system. 

## 2. Role of Microorganisms in Energy Regulation

### 2.1. SCFAs

The human gastrointestinal tract (GI) is the site of digestion and absorption of macro- and micronutrients for the host [27], as well as the maintenance of other processes necessary for proper systemic homeostasis (i.e., immunity [28]). Many mono- and disaccharides are naturally absorbed in the small intestine through passive and active diffusion and a myriad of transporters, where more complex carbohydrates arrive in the distal colon largely undigested [29]. Evidence has shown that the microbiota actively participates in host energy regulation from salvaging energy from otherwise undigested foodstuffs (i.e., fiber, lignin, pectin, starches) [30]. An early study in the 1970s demonstrated the ability of gut microbes to ferment various sources of indigestible carbohydrates, namely amylose, amylopectin and pectin [29]. Recently, the importance of *Bacteroides* species and their ability to degrade a wide variety of plant polysaccharides with a large array of glycoside hydrolases (GHs) and polysaccharide lyases (PLs) [31] has increased attention to describe the interaction between microbe derived nutrients and host biology. Compared to the human genome, the gut microbiome displays enrichments of gene sequences involved with polysaccharide, amino acid and polypeptide metabolism [32] when compared to previously sequenced microbial genomes. The sources of undigested carbohydrates include animal glycans [30], synthesized from other microbes and primarily, as components of dietary fiber such as cellulose, xyloglucan, mannan and xylan [31]. The principle byproducts of gut microbial fermentation are primarily SCFAs [29] and gases such as CO_2_, CH_4_ and H_2_ [27] which can influence both the host and microbe physiology.

SCFA’s are volatile fatty acids produced as a byproduct of microbial fermentation of undigested food stuffs in the large intestine and can supply roughly 10% of total energy for the host [33]. The most abundant SCFAs are acetic acid (C2), propionic acid (C3) and butyric acid (C4) [34]. SCFAs can stimulate the release of neuropeptides, small molecules, and other gut hormones from enteroendocrine cells [35] and are largely at proportions of 60:20:20 for acetate, propionate, butyrate, respectively [36]. Recently, it has been shown that SCFA dysbiosis is involved in several host inflammatory-based diseases, namely, ulcerative colitis and obesity [37]. SCFA are also derived from branched-chain amino acids producing valerate, formate, isobutyrate, isovalerate, caproate and 2-methyl-butyrate are also produced in smaller amounts [38]. In the gut lumen, acetate occurs at the highest concentrations and is central to lipid metabolism as a component of acetyl-coenzyme A [34]. Acetate is easily absorbed within the gut and is transported to the liver, where it is a primary substrate for cholesterol synthesis [36]. In 2016, Perry et al. [39] demonstrated in rats, increases in acetate promoted glucose-stimulated insulin secretion (GSIS), hyperphagia and increased ghrelin section via activation of the parasympathetic nervous system, that was not seen during a vagotomy; suggesting increased acetate production from microbial dysbiosis and further parasympathetic activation, may offer targets for treatment of obesity. In 2017, Dalby et al. [40] demonstrated increases in acetic, butyric, and propionic acid concentrations in chow-fed mice compared to mice fed a refined low- or high-fat diet, illustrating the importance of diet selection in examining the role of the gut microbiota and obesity. In 2013, Ridaura et al. demonstrated transplanting fecal material from a human twin pair discordant for obesity into GF mice, lean recipient mice had significant increases in cecal concentrations of propionate and butyrate when compared to obese transplanted mice. Interestingly, co-housing transplanted obese and lean mice together, also reported significant increases in propionate and butyrate [24]. Propionate is also a component of metabolism, serving as an odd chain fatty acid and is known to be used a substrate for gluconeogenesis [36]. Butyrate naturally occurs in animal fats and plant oils and is preferentially used by colonocytes as an energy source [34] and is believed to play a role in gut barrier function by improving the integrity of tight junctions [41]. In 2009, Gao Z. et al. [42] demonstrated in mice on a HFD, that butyrate improved host insulin sensitivity and potentiated energy expenditure, largely from improvements in mitochondrial function and biogenesis. Butyrate also has some anti-inflammatory properties and regulates gene expression, differentiation, and apoptosis [43]. One of the most abundant anaerobes in the human gut, with proportions around 5–8% of total bacteria in feces is *Faecalibacterium prausnitzii*, a member of the *Firmicutes* phylum [44] and plays a pivotal role in a healthy gut [45]. *F. prausnitzii* is known to produce butyrate and be involved with other anti-inflammatory agents, namely stimulating expression of anti-inflammatory cytokine interleukin 10 (IL-10) and reducing the secretion of proinflammatory IL-8 and, strongly inhibiting nuclear factor kappa-light-chain-enhancer of activated B cells (NF-κB) in cancer cells [45]—though full fermentation and butyrate pathways remain undefined. Notably, concentrations of *F. prausnitzii* are decreased in Crohn’s disease, inflammatory bowel disease (IBD) and type-2 diabetes [45]. Interestingly, our lab has shown increases *F. prausnitzii* concentrations in the mouse feces following exercise treatment [46]. Another powerful byproduct of microbial fermentation is H_2_, known to influence microbial interactions to impact selection of bugs within this complex community [27]. H_2_ has been shown to inhibit microbial fermentation within the gut [47]. 

### 2.2. Studies Investigating SCFA in Humans

Diet has been shown to be an important modulator of intestinal SCFA concentrations. In 2010, De Filippo et al. [48] compared the impact of diet on microbial community selection in children from Europe and a rural village in Burkina Faso (BF) and found a significant enrichment in Bacteroidetes and depletion in Firmicutes phyla in the BF cohort. Furthermore, significant increase in total SCFA production, with increases in acetic, propionic, butyric and valeric acids were observed in BF children [48]. In 2011, Arumugam et al. [8] described differences between gut enterotypes in 39 individuals using sources of energy for fermentation; *Bacteriodes* enterotype preferentially used carbohydrates and proteins, where *Prevotella* with co-occurring *Desulfovibrio* spp., acts to degrade mucin glycoproteins distributed across the mucosa. Still, the *Ruminococcus* enterotype, with its associated co-occurring species *Akkermansia* is also known to degrade intestinal mucins [8]. Wu et al. [49] confirmed the presence of *Bacteroides* and *Prevotella* enterotypes that remained stable over a 10-day study, but displayed changes in microbial composition within 24 h. Thus, only long-term host dietary practices were correlated with enterotype clustering [49]. In contrast, David et al. [11] reported on dietary-induced alterations of the gut microbiome in humans where, plant-based diets increased abundance of Firmicutes known to metabolize plant polysaccharides and animal-based diets increased abundance of bile-tolerant microbes [11]. Furthermore, reductions in acetate and butyrate in predominately animal-based diets were reported [11]. Butyrate has also become a novel substrate for use in clinical populations. In a recent clinical trial [50] in type-2 diabetic patients, butyrate + inulin supplementation was shown to reduce diastolic blood pressure (*p* < 0.05) when compared to the placebo group. Notably, butyrate combined with inulin demonstrated significant reductions in fasting blood sugar (*p* = 0.049) and waist-to-hip ratio (*p* = 0.020), as well as increased GLP-1 (*p* < 0.05), supporting supplementation of inulin in patients with T2D may reach clinical relevance [50]. In the same clinical trial, butyrate + inulin supplementation improved host inflammatory status, with reductions in tumor necrosis factor-α (TNF-α, *p* < 0.05) mRNA expression, highly sensitive C-reactive protein (hs-CRP) and malondialdehyde (MDA) levels (*p* < 0.05) [51]. Consistent with these findings, Zhao et al. [26] found increases in acetate and butyrate following an intervention with a diet high in non-digestible carbohydrates in type-2 diabetes (T2D) patients. 

### 2.3. Increased Energy Harvesting

As previously stated, work by Jeff Gordon’s lab demonstrated alterations in key microbial species and an increase in energy harvesting [2,18,19] in obese mice. In an early paper, Backhed et al. [9] showed the differences between GF and conventionalized mice, where conventionalized mice had a 60% increase in body weight and insulin resistance resulting from *de novo* hepatic lipogenesis, where Fiaf inhibited LPL activity facilitating triglyceride accumulation in the adipocytes. Notably, this was seen in both males and females as well as in different strains of mice. This data supports the role of the microbiota in host energy regulation in rodents. In 2007, Backhed et al. [22] showed GF mice were protected against obesity, with increases in skeletal muscle and hepatic AMPK expression when fed a Western-style, diet. Furthermore, GF knockout mice of *Fiaf*-/- showed decreases in the PGC-1α resulting in decreased fatty acid oxidation in liver and gastrocnemius muscle [22]. In an aforementioned study, Ridaura et al. [24] examined the microbiota component of human twins discordant for obesity, by transplanting fecal material from obese/lean co-twins into GF mice. Transplanted mice with an obese co-twin fecal microbiota, increased body mass and adiposity when compared to lean co-twin communities. Interestingly, co-housing transplanted lean and obese mice together prevented increases in body weight and adiposity seen in obese mice, where the reverse (e.g., obese to lean) was not seen [24]. This study demonstrates the transmissible effects of an altered microbiota to modulate host biology. Collectively, these results demonstrate the role of the gut microbiota and its byproducts in regulating host energy homeostasis. Underlying mechanisms connecting the microbiota, SCFAs and their receptors (GPR41 and GPR 43) are currently under investigation.

In 2015, den Besten et al. [52] demonstrated in mice the ability of SCFAs to protect against HFD-induced obesity via reductions of peroxisome proliferator-activated receptor-γ (PPARγ), which increased mitochondrial expression of uncoupled protein 2 (UCP2) and AMP/ATP ratio. These beneficial effects were reported in both adipose and hepatic tissue, suggesting SCFAs may offer effect therapeutics at selectively modulating PPARγ. Backhed, Ding et al. 2004 [9] suggest the ability of the microbiota to regulate *Fiaf* activity via the transcription factors carbohydrate response element binding protein (ChREBP). Another transcription factor that regulates hepatic lipogenesis is sterol response element binding protein (SREBP) [53]. Binding of SCFAs to G protein-coupled receptor (GPCRs) can also affect host energy regulation via neuropeptides (PYY) [54]. Acetate has been shown to stimulate adipogenesis and mitochondrial biogenesis via GPR43 [55] in brown adipose tissue (BAT). Samuel et al. [56] showed the microbiota to regulate GPR41 to affect host adiposity. Acetate and propionate preferentially bind to GPR43, whereas butyrate and propionate preferentially bind to GPR41 [57]. Collectively, these studies demonstrate in animal models as well as in humans, the importance of SCFAs to modulate host energy regulation. However, the sources of the various SCFAs are largely unknown and thus, require further investigation to understand the complex mechanisms connecting the microbiota, host energy regulation and its impact in modulating host inflammatory response. 

### 2.4. Dietary Protein and Energy Regulation

In addition to the modulating effects of dietary fiber on the gut microbiota, dietary protein intake has also been shown to play a role in regulating gut barrier integrity and potentially host inflammatory activity [58]. Absorption of endogenous and dietary protein is usually an efficient process [59] and primarily takes places throughout the small intestine; however, larger amounts (6–18 g/day [60]) may reach the colon and act as substrates for microbial degradation. As the host increases dietary protein consumption, gut microbial communities shift from using carbohydrates to protein sources for fermentation [61] that importantly, extend colonic transit times and pH [62]. Protein and amino acid fermentation is largely seen as deleterious to the host [63], with the production of hydrogen sulfide, reactive oxygen species (i.e., *N*-Nitroso compounds) and ammonia; all known to elicit toxic effects to the gut lumen [64]. Accordingly, in 2014, Liu et al. demonstrated decreases in *Faecalibacterium prausnitzii, Clostridium coccoides* and *Clostridium leptum* abundance in rats following a diet high in protein/low carbohydrate diet [65]. In contrast, tryptophan catabolites have recently gathered attention at modulating gut microbial communities and may modulate host immune response, enhance intestinal epithelial barrier function, and exert anti-inflammatory responses [58]. Tryptophan is one of the essential amino acids and thus, cannot be synthesized by the host. Tryptophan catabolites such as tryptamine, indolelactic acid (ILA), indoleacetic acid (IAA) and tryptamine can impact gut barrier function by modulating aryl hydrocarbon receptor (AHR) activity, whereas other tryptophan catabolites such as indole and indolepropionic acid (IPA), can modulate pregnane X receptor (PXR) activity to regulate the intestinal epithelial barrier [58]. Furthermore, indole can modulate host metabolism through the production of GLP-1 [58]. 

### 2.5. Studies Investigating Increased Dietary Protein Intake in Humans

In 2011, Russell et al. investigated the role of diets high in protein and low in carbohydrates on colonic health and demonstrated reductions in SCFA (namely butyrate) concentrations as well as reductions in *Roseburia/Eubacterium rectale* abundance, suggesting long-term adherence to diets high in protein but low in carbohydrates may not be beneficial to colonic health [66]. In contrast, Windey et al. found in a randomized cross-over study design of 20 healthy subjects that protein fermentation did not elicit gut toxicity [67]. For a more thorough review of dietary protein and its role on colonic health, the reader is directed to [68]. Future studies are needed to determine the role of dietary protein intake, in modulating gut microbial activity and its role in host colonic health.

## 3. LPS, Inflammation and Dietary Lipids

Bacterial LPS is the major component of the outer surface membrane present in most Gram-negative bacteria [69]. LPS is comprised of a distal polysaccharide (also termed O-antigen) and a nonrepeating “core” oligosaccharide region that is anchored in the outer bacterial membrane by a lipophilic, carbohydrate lipid moiety termed lipid A [69,70]. The lipid A component (endotoxin) is the primary immunostimulatory center of LPS and is detected by toll-like receptor 4 (TLR4) of the innate immune system [71,72]. Lipid A activation of TLR4 initiates the biosynthesis of inflammatory mediators, such a tumor necrosis factor alpha (TNF-α) and interleukin 1 beta (IL1-β) [73,74], and subsequent adaptive immune responses following activation of the downstream target nuclear factor κB (NF-κB) [75]. It is understood that the co-receptor CD14/TLR4 complex is required for LPS-induced secretion of proinflammatory cytokines as well [76]. Together, both the CD14/TLR4 complex and NF-κB activation are required to induce the immunostimulatory effects of LPS; CD14 deficiency prevents the innate immune response to bacterial LPS in mice and the secretion of proinflammatory cytokines in murine macrophages [77], while the inhibition of NF-κB prevents LPS-mediated TLR4 induction in monocyte cells [78]. This prevailing LPS-derived inflammatory pathway has long been investigated in the context of inflammatory disorders ranging from metabolic syndrome (MetS) to IBD wherein chronic low-grade inflammation is a common feature. As such, dietary interventions are often investigated to assess either their restorative or disease contributing aspects. Considering the present review, emerging evidence suggests a synergistic and proinflammatory relationship between LPS and dietary lipids that follow impairments in intestinal permeability. Furthermore, LPS-induced inflammation in target tissues may in part be dependent on the gut microbiota.

### 3.1. Gut Permeability

Intestinal host-microorganism homeostasis can be defined as minimizing the adverse health effects of intestinal microorganisms, even during environmental perturbations [79]. These include shifts in microbial community structure, changes in host behavior (i.e., diet and physical activity) or overt pathogenic challenge. As such, multiple immunological barriers are in place to ensure mucosal homeostasis and to resolve inflammatory responses as they arise [79]. The onset of metabolic disease is often associated with low-grade chronic inflammation of the intestinal mucosa, which leads to a slow degradation of epithelial barrier integrity, however. This idea of the “leaky gut” was first termed by Gummesson et al. [13] who first established an association between visceral adiposity and gut leakiness. At present, studies have corroborated these findings and strongly suggest that dietary lipids indeed play a part in enhancing gut inflammation and permeability wherein concomitant shifts in microbial community structure follow [80,81]. 

#### 3.1.1. HFDs 

Recently, the findings of Lam et al. confirm that dietary fat profile contributes to either the improvement or degradation of gut barrier integrity [82]. Two cohorts of C57BL/6J female mice were used. The first comprised of four diet groups fed either a 60% high saturated fat (HFD-sat; 34% kcal), high n-6 PUFA (HFD-n6; 31% kcal), high n-3 PUFA (HFD-n3; 37% kcal) or a control diet (CTRL) for 8 weeks *ad libitum*. In the second, mice were first fed either a CTRL or HFD-sat diet for 8 weeks. CTRL groups were then given intraperitoneal injections of saline or high-dose resolvin D1 (a docosahexaenoic acid metabolite) and HFD-sat groups were given saline, low-dose resolvin D1, high-dose resolvin D1 or n-3 supplementation with fish oil; stool samples were collected at the end of the study. Transepithelial resistance of the colon was determined in an Ussing chamber by measuring change in potential difference. As expected, colonic transepithelial resistance was 26% lower in HFD-sat mice compared to controls; HFD-n6 and CTRL groups did not differ but HFD-n3 tended to increase transepithelial resistance. Bacterial DNA in mesenteric fat was assessed as a surrogate marker of bacterial translocation and HFD-sat trended 2-fold times higher relative to CTRL groups. Following fecal microbial analysis, it was found that OTUs associated with impaired gut barrier function included *Erysipelotrichaceae* and *Bilophila*, whereas obesity was linked to the presence of *Enterococcus* and *Lachnospiraceae incertae sedis* [82]. The genus *Bilophila* includes sulfate-reducing bacteria which produce dihydrogen sulfide (H_2_S). Previous reports have shown that dietary fat profile select for these microorganisms and are associated with subsequent changes in intestinal inflammation [83]. In the present study, the proportion of H_2_S-producing bacteria, primarily *Bilophila* and *Desulfovibrio*, rapidly increased (3-fold times) during the first 2 weeks of HFD-sat feeding and continued to increase overtime. In contrast, these bacteria remained stable in HFD-n6 mice whereas HFD-n3 feeding reduced their abundance; the abundance of H_2_S-producing bacteria was negatively correlated with transepithelial resistance as well [82]. In cohort 2, transepithelial resistance was significantly increased in a dose-dependent manner in HFD-sat animals given resolvin D1 (24–68%); fish oil supplementation also restored gut permeability to that of controls. Furthermore, high-dose resolvin D1 significantly ameliorated gut inflammation in HFD-sat animals as determined by quantification of F4/80^+^ cells. Likewise, fish oil reduced macrophage abundance by 72% compared to HFD-sat animals given saline [82]. Lastly, fish oil or high-dose resolvin D1 selected for *Bacteroides* and completely abolished the HFD-sat induced enrichment of *Desulfovibrio* [82]. These findings conclude that HFDs rich in saturated fatty acids (SFAs) enhance intestinal permeability and may select for H_2_S-producing bacteria. In contrast, supplementation with either fish oil or the DHA metabolite, resolvin D1, can rescue these effects and maintain epithelial barrier integrity despite excess consumption of SFAs.

Though the influence of dietary lipids on intestinal permeability is well known, a causal relationship between the degradation of epithelial barrier integrity and alterations in the gut microbiota in the context of diet-induced obesity remains to be defined. A recent report by Hamilton et al. sought to gain insight and elucidate key mechanisms by which HFDs challenge intestinal barrier function in both the small and large intestine [84]. 9–10-week-old male Wistar rats were fed a 45% HFD for 1, 3 or 6 week and gut barrier function was assessed *ex vivo* in an Ussing chamber. Both paracellular and transcellular pathways were measured as the flux of FITC-dextran 4000 (FD4) and horseradish peroxidase (HRP). Analysis revealed that the HFD increased paracellular permeability in both the ileum at week 1 and colon at week 3; paracellular permeability returned to control values by week 6, however. In contrast, a progressive and sustained increase in transcellular flux was observed in the cecum and colon of HF-fed rats compared to controls beginning at week 3. Histological analysis further showed a decrease in colon length, fecal weight, fecal and ileal crypt depth, and fecal goblet cells after week 1. At week 3, tight junction (TJ) protein zonula occludens-1 (ZO-1) increased in the ileum as well as caveolin-1 (CAV1; endocytosis and lipid raft protein) at all time points [84]. Furthermore, the HFD increased IL-1β gene expression as well as plasma levels of LPS-binding protein (LBP) over time. Interestingly, elevated plasma levels of LBP were not observed until week 3 as seen in fecal fat content and transcellular flux as well. 

Microbial analyses were then performed to determine regional and diet-induced differences of community structure. At the phylum level, the Firmicutes to Bacteroidetes ratio increased in both the ileum and cecum in HF-fed rats compared to controls. In addition, Deferribacteres was significantly increased in the cecum but not the ileum of HF-fed rats whereas Cyanobacteria was significantly greater in the ileum, but not the cecum, of chow-fed rats. At the genus level, the abundance of 13% of all bacterial genera detected in the ileum was significantly changed by diet; the most abundant genera in HF-fed rats were *Lactococcus* and *Corynebacterium* whereas an unclassified genus of the *Clostridiaceae* family was the most abundant in chow-fed rats. As expected, changes in abundance at the genus level were significantly larger in the cecum. The most abundant genera were *Prevotella* in chow-fed rats and *Bacteroides* in HF-fed rats. Lastly, correlational analyses showed that *Oscillospira* in the ileum was significantly correlated with small intestine paracellular permeability whereas *Butyricimonas* and an unclassified genus were significantly correlated with fecal transcellular flux. Moreover, the large intestine transcellular pathway, but not the paracellular pathway, was highly correlated with adiposity, body weight and LBP [84]. The above findings highlight a time-dependent and region-specific interaction between the obese phenotype, alterations in epithelial transport pathways and alterations in the gut microbiota. The results of the present study also describe the early onset effects of HFDs. In short, HFDs appear to primarily affect transcellular permeability in the large intestine and potentiate ileal inflammation. Furthermore, systemic infiltration of LPS may be mediated by fecal fat content and enhanced transcellular flux. As discussed by the authors however, amount of fat may not explain the observed increase transcellular flux as CAV1 gene expression preceded these changes [84]. Finally, principle genera of the gut microbiota are correlated with epithelial pathway permeability. 

#### 3.1.2. Microbial Secretory Metabolites

As the role of the gut microbiota in regulating host intestinal permeability becomes increasingly defined, ecological, and correlational analyses are beginning to highlight putative molecular mechanisms by which such alterations in host barrier integrity are conferred. Much attention has been placed on microbial-derived metabolites, namely LPS and SCFAs, to understand the dynamic yet integrated nature of microbial and host physiology. At present, emerging evidence suggests that extracellular vesicles (EVs), a functional metabolite secreted from gut microorganisms, may significantly influence gut permeability as well. EVs are lipid bilayer structures secreted from both Gram-negative and positive bacteria [85,86] and are composed of proteins, lipids, nucleic acids, LPS and other virulence factors [87,88,89]. EVs transfer genetic material and proteins to the host and interact directly with immune and epithelial cells [90]. In this context, EVs can be considered functional units of the gut microbiota that mediate host-pathogen interactions. The extent of beneficial or harmful effects that EVs transfer to the host may be dependent on the microbe of origin, however. Of note, Chelakkot and colleagues have recently described the intestinal regulatory effects of EVs derived from *Akkermansia muciniphila* [91]. EVs were isolated from cultures of *A. muciniphila* and C57BL/6 mice were fed a 60% HFD for 12 weeks. Both *in vitro* and *in vivo* assays of permeability were employed using (FITC)-dextran in Caco-2 cells and in fasted mice following the dietary intervention. Lastly, EVs were isolated from fecal samples of healthy and T2D patients by ultracentrifugation. In humans, Firmicutes dominated in feces of T2D patients whereas Proteobacteria, Bacteroides and Verrucomicrobia had a greater relative abundance. Interestingly, it was found that EVs had a distinct microbial composition relative to fecal contents; as expected, EVs from *A. muciniphila* were detected only in healthy controls. To elucidate the role of EVs isolated from *A. muciniphila* (AmEVs), these were compared to *E. coli* EVs (EcEVs) given that pathogenic strains of *E. coli* are associated with intestinal inflammation and enhanced intestinal permeability [92,93,94]. Transmission electron microscopy determined that both AmEVs and EcEVs were spherical in shape, had lipid bilayers and similar diameters (40–60 nm). Though the physical characteristics remained the same, SDS-PAGE analysis showed that protein composition varied extensively [91]. As a proof of concept, additional mice were administered AmEVs conjugated with Cy7 via oral gavage and visualized *in vivo* using near-infrared imaging. After 6 h, it was revealed that AmEVs spread throughout the mouse body. *Ex vivo* imaging of harvested organs further confirmed that AmEVs translocated to and remain isolated in the large intestine. In mice undergoing the dietary intervention, AmEVs were administered that last 2 weeks of the study every other day via oral gavage. Interestingly, AmEV feeding significantly reduced body weight gain in HF-fed mice and attenuated HFD-induced increases in intestinal permeability as indicated by a reduction in serum FITC-dextran concentration. Moreover, treatment of AmEVs reversed the HFD-induced suppression of occludin, ZO-1 and claudin-5 protein expression [91]. 

*In vitro* assays in Caco-2 cells were then performed to model metabolic endotoxemia as seen in metabolic disorders to elucidate a mechanism by which EVs regulate TJs. In short, Caco-2 transwell monolayer cultures were treated with LPS alone, EcEVs or AmEVs [91]. In agreement with the above findings, LPS-induced decrease in transepithelial resistance, increase in FITC-dextran permeability and decrease in occludin protein expression were rescued by AmEVs; no significant changes were observed with co-treatment of EcEVs. Mechanistically, it was also found that AmEVs induced phosphorylation of AMPK with a concomitant increase in occludin protein expression in a dose-dependent manner [91]. To confirm the role of AMPK, cells were then cotreated with AmEV and AMPK inhibitor compound C; inhibition of AMPK abolished AmEVs’ ability to improve LPS-induced degradation of barrier integrity and to induce occludin expression [91]. Taken together, these findings highlight the potential role of EVs in regulating host barrier integrity and suggest that the degree of benefit or harm to the host is dependent on the microbe of origin. Furthermore, EVs vary extensively in their molecular constituents between different microbes despite being similar in shape and size. EVs derived from *A. muciniphila* appear to attenuate both HFD and LPS-induced gut barrier impairments and may regulate TJs through activation of AMPK, a kinase response for TJ re-assembly and stability [95,96,97].

#### 3.1.3. Gut Permeability in Humans

As exemplified in the above studies, assessment of the impact of dietary lipids on gut barrier integrity has been achieved using primarily animal models. Yet, recent findings in humans corroborate the deleterious effects of dietary lipids on intestinal inflammation and permeability. For instance, Morales et al. [98] employed a unique experimental approach to define the acute impact of fat malabsorption and fiber on colonic barrier function using the common drug Orlistat (tetrahydrolipstatin), an inhibitor of pancreatic lipase that is used in the treatment of obesity and dyslipidemia [99]. Forty-one asymptomatic male and female volunteers aged 18–40 years with healthy BMIs were included and divided into four groups: control, Orlistat, prebiotic (oligofructose) and Orlistat/prebiotic. Daily intake of fat was standardized to about 60 g per day for one week without treatment and one week with treatment; fecal samples were taken over a 72 h period at both time points. Urinary concentrations of lactulose/mannitol and sucralose were used as markers of proximal and distal intestinal permeability, fecal calprotectin was measured to assess colon inflammation and antioxidant activity of fecal water was determined by the ferric ability of plasma (FRAP) assay [100]. As expected, fecal calprotectin levels were significantly increased in the group given Orlistat, but this response was prevented when individuals were concomitantly given the prebiotic. Likewise, Orlistat administration significantly decreased antioxidant activity but this decrease was prevented with co-administration of the prebiotic. No changes in urinary excretion of lactulose, mannitol, and sucralose as well as lactulose/mannitol or lactose/sucralose ratios were found. Lastly, changes in alpha diversity remained non-significant and analysis of beta-diversity showed that samples did not cluster according to treatments though high interindividual variability was reported. 

In a cross-sectional study by Mokkala et al. [101], fecal and serum samples from 100 overweight Finnish women in early pregnancy were collected along with three-day food diaries to examine associations between serum zonulin concentrations, microbial composition, and diet. Of note was the finding that low zonulin levels in serum were associated with lower abundances of the genera *Bacteroides* and *Blautia* as well as with a higher abundance of the genus *Faecalibacterium*. Furthermore, *Faecalibacterium prausnitzii* had a higher abundance in the low zonulin group; no differences in the Bacteroides to Firmicutes ratio were found between low and high zonulin groups [101]. Likewise, *Faecalibacterium prausnitzii* was negatively and *Blautia* positively associated with serum zonulin concentrations. Analysis of diet showed significant differences in absolute nutrient intakes between high and low zonulin groups in which individuals in the low zonulin group consumed more protein, total PUFAs, n-3 PUFAs and dietary fiber. Linear regression analysis of nutrient intake further revealed a significant inverse relation between serum zonulin concentration and absolute intakes of protein, total PUFAs, n-6 PUFAs as well as several micronutrients. Interestingly, protein intake was found to be a significant factor in predicting serum zonulin when energy-yielding nutrients were included in an adjusted multiple linear regression analysis while no significant associations were found for any single micronutrient. 

Most recently, Genser and colleagues [102] employed an innovative study design to investigate jejunal permeability in severely obese and non-obese patients using both *in vivo* and *ex vivo* analyses where proximal jejunum samples were taken from obese individuals during Roux-en-Y gastric bypass (RYGB) surgery and from non-obese controls who underwent a pancreaticoduodenectomy or gastrectomy. Immunofluorescence showed a significant reduction of occludin and tricellulin expression, a TJ protein localized in both tricellular and bicellular TJs [103]. *Ex vivo* analysis of permeability using FITC-labeled sulfonic acid or dextran (4 kDa and 10 kDa) in an Ussing chamber demonstrated that jejunal permeability values were more heterogenous in the obese patients but no significant differences were found relative to controls which agreed with urinalysis of lactitol/mannitol ratios. Similarly to Mokkala et al. [101], fasting serum levels of zonulin were elevated in obese patients as well as LBP. Though no statistical differences in jejunal permeability were found between obese patients and non-obese controls, obese patients showed varying levels of systemic low-grade inflammatory markers and fecal calprotectin in which several were positively correlated with values of permeability such as haptoglobin and C-reactive protein [102]. Human Caco-2/TC7 intestinal cells exposed to lipid micelles demonstrated no change in transepithelial resistance but an enhanced permeability to large-size molecules (≥4 kDa) and a reduction in tricullulin expression. Furthermore, micelles containing digestion products of triglycerides (“postprandial micelles”) significantly increased paracellular permeability but “interprandial micelles” containing biliary products had no effect [102]. Likewise, exposure to postprandial lipid micelles significantly increased permeability to 4 kDa FITC-dextran in jejunal explants from both obese and control patients. However, postprandial micelles had a far more potent effect on permeability in obese explants with an increase of +68% mean fold difference relative to baseline compared to +45% in controls. 

In all, these human studies demonstrate that an acute exposure to large amounts of dietary lipids induce inflammation and reduce antioxidant activity in the colon that precede changes in the gut microbiota. In contrast, adequate intake of dietary fiber may attenuate colonic inflammation in the presence of excess dietary lipid consumption. In addition, serum zonulin concentrations may be indicative of microbial shifts and that lower concentrations may promote the enrichment of *F. prausnitzii,* a bacterium emerging as a potent probiotic [104]. Moreover, protein intake may be more of a potent predictor of serum zonulin concentrations which perhaps highlights the importance of satiety and total caloric consumption regarding management of adiposity. Lastly, severely obese individuals have increased serum zonulin concentrations and are more susceptible to gut barrier degradation when exposed to dietary lipids despite having no difference in intestinal permeability relative to non-obese individuals. 

#### 3.1.4. Fusobacterium and Colorectal Cancer

Inflammatory disorders and changes in microbial community structure often occur in tandem. As such, characterization of the whole gut microbiota in diseased states is performed to capture a more complete view of the gut ecological landscape. In the context of IBD, early studies have demonstrated an association with *Fusobacterium* species, a known risk factor for colorectal cancer development [105,106,107]. First articulated by Sears and Pardoll [108], this concept of an “alpha-bug” illustrates that select members of a microbial community can indeed remodel the whole of the microbiome to drive proinflammatory immune responses and colonic epithelial cell dysfunction leading to cancer. In support of this hypothesis, two early concurrent studies showed *Fusobacterium* to be enriched in the tumor microenvironment of the colonic mucosa [109] and that specifically *Fusobacterium nucleatum* is prevalent in human colorectal carcinoma [20]. 

To date, there are four primary mechanisms of action by *F. nucleatum* in CRC and have recently been reviewed in depth [110]. To summarize, *F. nucleatum* may first demonstrate anti-immune effects against tumors through increased infiltration of CD11b^+^ myeloid immune cells which enhances tumor development and angiogenesis, increased tumor-associated macrophages (TAMs) which inhibit CD4 T cells, increased angiogenesis and inhibition of natural killing (NK) cell cytotoxicity. Second, *F. nucleatum* possesses distinct virulence factors that include self-enrichment through binding to the disaccharide tumor marker D-galactose-β(1-3)-N-acetyl-D-galactosamine (Gal-GalNAc) which is present in cancer cells and in rectal mucous of colon cancer patients [111], epithelial and endothelial adhesion and invasion by adhesion A protein (FadA), and EVs which act as a delivery system for additional virulence factors. Third, *F. nucleatum* metabolizes peptides and amino acids for nutrients where the byproducts are chemoattractants of myeloid cells [112]. Lastly, *F. nucleatum* upregulates the expression of miR-21, a microRNA posited to play an important role in chronic intestinal inflammation and the progression of colitis-associated CRC [113]. Thus, *F. nucleatum* possesses an extensive repertoire of virulent tactics that promote the induction of host proinflammatory responses. Despite its virulence, dietary interventions may attenuate CRC susceptibility by *F. nucleatum*. Specifically, recent studies in humans have shown that diets rich in whole grains and dietary fiber are associated with a lower risk of *F. nucleatum*-positive CRC [114] while diets low in fiber but high in fat are associated with an increase in *F. nucleatum* [115] which further corroborates the utility of dietary fiber in disease prevention by manipulation of the gut microbiota. 

### 3.2. White Adipose Tissue

The role of white adipose tissue (WAT) extends far beyond energy storage. WAT acts as an endocrine organ, secreting factors (adipokines) required for a diverse array of host functions including host immunity [116]. In metabolic disorders such as obesity, there is an expansion of WAT and an increased inflammatory tone [117]. The mechanisms of this inflammatory response may include both oxidative and endoplasmic reticulum (ER) stress among others, as well as dysregulation of free fatty acid (FFA) flux [118,119]. FFAs can serve as a TLR4 agonist [120], activating proinflammatory pathways, stimulating the influx of cytokines and thereby driving macrophage accumulation and inflammation [121,122,123,124,125]. In addition, stimulation with LPS induces lipolysis, insulin resistance [126] and secretion of proinflammatory adipokines including the chemokine monocyte chemoattractant protein (MCP-1) [127], a key regulator of macrophage recruitment. Fatty acid type also plays a critical role in WAT homeostasis and diets rich in SFAs are associated with increased WAT inflammation [128]. Studies have shown that mice fed a HFD have an increased expression of MCP-1 mRNA in adipose tissue and elevated MCP-1 plasma protein levels [129]. Recent findings of Cullberg et al. [130] further indicate that LPS and dietary fatty acids induce WAT inflammation, but through different cell types involving MCP-1 in 3T3-L1 murine adipocytes and THP-1 human macrophages. Specifically, LPS stimulated MCP-1 expression in a dose-dependent manner while none of the three tested FFA (oleic, elaidic and palmitic acid) affected MCP-1 mRNA and protein expression in adipocytes. Moreover, there were no additive or anti-inflammatory effects of any investigated FFA. In contrast, incubation with palmitic acid (PA) increased MCP-1 mRNA 1.8-fold times in macrophages with elaidic and oleic acid having no effect. Remarkably, expression of TLR4 was 96.6% higher in macrophages than in adipocytes [130]. These results suggest that LPS induces WAT inflammation through the upregulation of MCP-1 in adipose tissue, while SFAs stimulate proinflammatory macrophage responses directly, perhaps due to the higher TLR4 expression levels. 

As LPS is a microbial-derived ligand for TLR4, the impact of the gut microbiota on host WAT homeostasis must also be considered as it is well established that alterations in the gut microbial ecology can modulate host innate immunity and metabolism [131]. In a recent study, Caesar et al. [132] demonstrated how SFA in lard altered the gut microbial ecology to promote obesity and WAT inflammation through TLR signaling and MCP-1. Eleven to 14-week-old mice were kept on a 45% HFD comprised of either lard or menhaden fish oil for 11 weeks. 24% of the variability in microbiota composition was due to fat source and alpha diversity decreased in mice fed lard. Enrichment of distinct species was also observed in animals fed their respective diets. Specifically, the genera *Bacteroides, Turicibacter* and *Bilophila* increased in lard-fed mice. In contrast, *Bifidobacterium* and *Adlercreutzia* (Actinobacteria); *Lactobacillus* and *Streptococcus* (lactic acid bacteria); *Akkermansia muciniphila* (Verrucomicrobia); Alphaproteobacteria and Deltaproteobacteria increased in mice fed fish oil [132]. No significant differences in systemic concentration of LPS were found between both groups, though systemic activation of TLR4 was enhanced by serum from mice fed lard. Furthermore, mice lacking the TLR adaptor molecule myeloid differentiation primary response 88 (MyD88) were protected against diet-induced obesity. WAT inflammation was elevated in lard-fed mice as indicated by an increase in crown-like structures (CLS), representing enhanced macrophage recruitment, and the accumulation of CD45+ leukocytes; this response was blunted in both MyD88 ^-/-^ and TIR-domain-containing adapter-inducing interferon-β (Trif) ^-/-^ mice for both CLS and CD45+ leukocytes, as well as in GF mice for CLS only but trending downward for CD45+ cells [132]. Lastly, WAT MCP-1 expression was higher in CONV-R lard-fed mice compared to GF mice; this response was also blunted in both MyD88 ^-/-^ and Trif ^-/-^ mice. Taken together, these results show that the type of dietary fat is a major driver in microbial composition and diversity, and that the gut microbiota contributes to HFD-induced WAT inflammation through enhanced adipocyte expression of MCP-1 in a MyD88-Trif-TLR4-dependent manner. These findings also confirm that diets rich in SFAs are associated with increased intestinal absorption of microbial factors (i.e., LPS) as evidenced by TLR4 activation in systemic circulation.

### 3.3. Liver

Consumption of HFDs rich in SFAs are a risk factor for non-alcoholic fatty liver disease (NAFLD) which is characterized by an accumulation of lipids in hepatocytes in the absence of excessive alcohol intake [133]. NAFLD can further be divided into two primary categories: non-alcoholic fatty liver (NAFL) and non-alcoholic steatohepatitis (NASH). While both achieve hepatic steatosis, the former occurs without inflammation or injury and the latter is characterized by the presence of inflammation and necrosis [134]. Innate immune responses play an essential role in the pathogenesis of NASH where the initiation of LPS-TLR4-mediated proinflammatory signaling cascades in hepatocytes, namely Kupffer cells, lead to hepatic inflammation, injury, and fibrosis [135]. In addition, increases in SFA consumption also contributes to NASH development [136,137] through mechanisms including ER stress, reactive oxygen species (ROS) accumulation and mitochondrial dysfunction [138]. As such, the interplay between LPS, SFA and the induction of hepatic inflammation is complex given the diversity of immunological, metabolic, and microbial-derived factors involved.

#### 3.3.1. Total Fat Content 

Given the potent proinflammatory effects of SFAs, much emphasis has been placed on the Mediterranean diet as a healthful alternative due to its high content of monounsaturated fatty acids (MUFAs) primarily from olive oil [139]. In stark contrast to this notion however, the recent findings of Meidan and colleagues suggest that olive oil may exacerbate liver inflammatory damage [140]. Six-week-old C57BL/6J male mice were fed a 60% HFD for 8 weeks containing palm stearin (PS) or olive oil, as well as a low 16% fat diet. Following the 8 weeks dietary intervention, animals were fasted for 12 h, treated with a single intraperitoneal injection of LPS and sacrificed 6 h thereafter. Although LPS treatment resulted in a trend of losing fat in the liver and hypoglycemia in all diet groups, plasma hepatic enzyme (SGPT, ALT and SGOT, AST) and insulin levels were significantly elevated in both high-fat groups injected with LPS; LPS had no effect on the low-fat diet group and no significant differences were observed between both HFD groups given LPS [140]. Combined treatment of LPS with both HFDs suppressed gluconeogenesis as well. Liver iNOS and IL-6 mRNA levels were significantly elevated in all three diet groups given LPS and PS plus LPS showed the greatest increase in iNOS mRNA expression. In addition to the dietary intervention, a fatty acid loading-gavage feeding protocol was implemented using 5–6 weeks old male C57BL/6J mice consuming a standard chow diet. Mice were force fed olive oil via gavage, and/or injected with LPS following an overnight fast and sacrificed 6 h thereafter [140]. Interestingly, blood glucose and triglyceride (TG) levels were reduced in animals receiving the oil gavage and LPS compared to acute individual treatments. In contrast, serum FFAs were significantly elevated in groups given olive oil and LPS [140]. Taken together, these findings conclude that HFDs of either MUFAs or SFAs may potentiate LPS-induced liver inflammation and hyperinsulinemia, perhaps by the induction of lipolysis and a subsequent increase in FFA flux, and that total fat content rather than type is a driver of inflammation. 

#### 3.3.2. Ceramide De Novo Synthesis and PA 

Though the above findings corroborate the impact of total fat content on liver inflammation, multiple complex inflammatory mechanisms remain at play and SFAs still pose a significant threat to hepatocyte homeostasis. Of note, Li et al. investigated the hepatic inflammatory response of SFA in combination with LPS in male low-density lipoprotein receptor deficient (LDLR^-/-^) mice and *in vitro* in a mechanistic approach to elucidate the synergistic effects of LPS and SFA [141]. Mice were fed an HFD with high or low PA content (HP-HFD and LP-HFD) for 20 weeks where half were subsequently injected with LPS during the last 12 weeks of the dietary intervention. Primary mouse hepatocytes and murine macrophage RAW264.7 cells were treated with LPS derived from *E. coli* and PA. Results showed that the HP-HFD in combination with LPS enhanced F4/80 expression more than the HP-HFD or LPS alone in liver tissue. Likewise, IL-6 protein expression in hepatocytes and mononuclear cells increased the most with the HP-HFD and LPS than either alone as determined by immunohistochemistry; the combination of the LP-HFD and LPS did not increase IL-6 protein expression to the same degree and LPS alone had no effect [141]. In hepatocytes *in vitro*, LPS stimulated both IL-6 secretion and gene expression while PA alone had no effect. In contrast, PA enhanced LPS-induced IL-6 mRNA expression [141]. In addition, the expression of proinflammatory genes involved in the TLR signaling pathway were significantly upregulated with both LPS and PA in tandem including MCP-1, Cox-2 and IL-1β [141]. As shown by Cullberg et al. above in adipocytes [130], Li et al. also demonstrated a crosstalk between hepatocytes and macrophages in which the extent of IL-6 and TNF-α production was the most enhanced when both cell types were cocultured in LPS plus PA [141]. Furthermore, inhibition of FFA receptors GPR40 and CD36 by sulfo-N-succinimidyl oleat or GW1100 suppressed IL-6 and MCP-1 secretion induced by LPS or LPS plus PA. Interestingly, PA plus LPS markedly increased total ceramide (CER) and dhC16-CER production, an intermediate in de novo CER synthesis. Consequently, inhibition of serine palmitoyltransferase, an enzyme involved in a rate-limiting reaction of CER de novo synthesis, by myriocin inhibited the stimulatory actions of LPS or LPS plus PA on IL-6 secretion and gene expression [141]. Lastly, PA alone significantly induced hepatocyte apoptosis in a concentration-dependent manner while the addition of LPS to PA modestly reduced the degree of apoptosis [141]. Collectively, these findings posit a critical mechanism by which LPS and PA contribute to hepatocyte proinflammatory processes in alternate but converging pathways. Specifically, LPS triggers the activation of inflammatory signaling pathways involving TLR4 while PA contributes to enhanced hepatocyte inflammation through the actions of FFA receptors GPR40 or CD36. The activation of both pathways results in a strong inflammatory response and engage in a cooperative stimulation of CER de novo synthesis which further exacerbates hepatocyte inflammation [141]. The presence of LPS may attenuate PA-induced hepatocyte apoptosis and promote the continuation of proinflammatory cytokine production. 

#### 3.3.3. Butyrate Supplementation

In contrast to LPS, other microbial-derived metabolites pose a benefit to the host. Of note are SCFAs which are major end-products of microbial fermentation in the gut and primarily formed from carbohydrate, protein, and glycoprotein precursors by anaerobic bacteria [38]. Principal SCFAs are acetate, propionate and butyrate which provide sources of carbon and energy for host tissues [142]. These organic acids are absorbed through regional mucosas of the digestive tract [142] and have been shown to modulate host energy homeostasis through interactions between chemosensory enteroendocrine cells [143]. Much emphasis has been placed on butyrate due to its protective effects against HFD-induced obesity, insulin resistance and hepatic steatosis [144,145]. Until recently, the underlying mechanisms by which butyrate confers its beneficial properties have remained obscured. In an elegant study design, Ye and colleagues investigated the protective effects of butyrate against diet-induced NASH in methionine-choline deficient mice [146]. Eight-week-old male C57BL/6J were divided into four groups of animals fed either a methionine-choline-sufficient (MCS) or methionine-choline-deficient (MCD) diet for 6 weeks and given sodium butyrate (SoB) or a vehicle via gavage. SoB reduced MCD-induced hepatic steatosis and normalized MCD-induced upregulation of IL-1β, TNF-α and F4/80 mRNA levels in liver tissue; immunohistochemical analysis confirmed a reduction of F4/80^+^ cell infiltration in MCD mice supplemented with SoB. MCD also downregulated potent anti-inflammatory cytokines IL-4 and IL-10 but this response was reversed in MCD mice fed SoB. In addition, MCD increased mRNA expression of both TLR4 and its co-receptor CD14 but was normalized with treatment of SoB. Serum concentrations of proinflammatory cytokines, such as IL-1β, IL-6, TNF-α and Eotaxin, increased in MCD mice but reduced when supplemented with SoB. As seen in liver tissue, SoB also upregulated serum levels of IL-10 and IL-4 relative to MCD groups. Likewise, serum LBP levels were reduced in MCD mice fed SoB [146]. 

To assess the extent of butyrate supplementation on epithelial barrier integrity in the colon, claudin-1 and ZO-1 mRNA levels were assessed along with immunostaining. SoB reversed MCD-induced suppression of claudin-1 and ZO-1 mRNA levels, and stabilized TJ structures at the protein level as evidenced by an improved organization of ZO-1 localization [146]. Furthermore, upregulation of TLR2 and TLR4 mRNA expression in MCD groups was observed but administration of SoB suppressed this response as seen in the liver. In the context of the gut microbiota, butyrate treatment attenuated MCD-induced dysbiosis in which a marked decrease in the abundance of Firmicutes and Tenericutes as well as a significant increase in the abundance of Verrocomicrobia and Proteobacteria was observed in MCD groups supplemented with SoB. At the genus level, SoB supplementation in MCD groups reduced the abundance of *Rikenellaceae*_RC9_gut_group and *Lachnospiraceae*_NK4A136_group levels but markedly enriched *Parasutterella* and *Akkermansia*; MCD alone enriched species of the *Bilophila* and *Rikenellaceae* genera [146]. Following metabolomic analysis, results showed that butyrate supplementation increased levels SFAs (stearic and behenic acid), unsaturated FAs (oleic and linoleic acid) as well as squalene but reduced levels of arachidonic acid [146]. 

To further examine the benefits of the altered microbiota derived from butyrate supplementation, the investigators then conducted a correlation analysis of an interaction matrix between microbial genera, metabolites, liver, and gut impairment parameters [146]. It was found that both *Bilophila* and *Rikenellaceae* were positively correlated with TLR4 and TNF-α among other proinflammatory markers. In contrast, TLR4, TNF-α, F4/80, IL-6 and LBP among others were negatively associated with *Akkermansia*. Furthermore, arachidonic acid was negatively associated with *Akkermansia* while stearic acid was positively associated with *Sutterella* [146]. In sum, these results highlight several complementary mechanisms by which the SCFA butyrate ameliorates diet-induced NASH. To summarize the authors, these include alleviation of dysbiosis, restoration of the colon epithelial barrier, regulation of lipid metabolism, and the attenuation of both liver and systemic inflammatory responses [146]. Furthermore, strong correlations exist between markers of inflammation, FFA type and ecological changes in the gut microbiota.

#### 3.3.4. Bile Acids 

Bile acids (BAs) are amphipathic steroid molecules synthesized from perivenous hepatocytes which are stored and concentrated in the gallbladder. Following food consumption, ingestion-stimulated cholecytoskinin (CCK) secretion by enteroendocrine cells stimulates gallbladder contraction and bile release into the duodenum [147]. BAs activate pancreatic lipase and form bile salt micelles containing dietary lipids and lipophilic vitamins (A, D, E and K) which are then transformed into secondary BAs by the gut microbiota [147,148]. In the context of inflammatory disorders, BAs are ligands of several receptors including the nuclear farnesoid X receptor (FXR) [149] and takeda G protein-coupled receptor 5 (TGR5) [150]. Dysregulation of these receptors can cause NAFLD and therapeutic interventions involving these receptors have been explored extensively in a recent review [148]. For instance, hepatic FXR and TGR5 agonists are being developed as an adjunct treatment for NASH due to their ability to inhibit lipogenesis and hepatic fibrosis as well as promote energy expenditure in BAT and induce GLP-1 secretion. Studies also demonstrate that TGR5 exerts inflammatory control through the suppression of cytokine production following innate immune responses [151,152,153]. 

Given that the gut microbiota facilitates the production of secondary BAs, potent stimulators of these receptors, its role in maintaining the bile acid pool is of primary interest. For example, regulation of FXR is largely accomplished through both primary BAs and secondary BAs produced by the gut microbiota, namely chenodeoxycholic acid (CDCA), lithocholic acid (LCA), deoxycholic acid (DCA) and cholic acid (CA) (muricholic acid in rodents) [154,155]. Thus, dysbiosis as seen in NALD may alter the composition of the bile acid pool by reducing the abundance of principal genera capable of primary to secondary bile acid conversion such as *Bacteroides* and *Lactobacillus*, which possess bile salt hydrolases for bile acid deconjugation [156]. For example, one study has shown that *Clostridium leptum* abundance is reduced in NASH which is capable of metabolizing BAs [157]. Of note are the findings of Kakiyama et al. [158] who demonstrated that total fecal BAs are significantly lower in advanced cirrhotic patients compared to healthy controls and those diagnosed with early cirrhosis. In addition, cirrhotic patients have a significantly lower percentage of fecal secondary BAs and ratios of primary to secondary BAs are highest in advanced cirrhosis. Furthermore, a significantly higher abundance of *Enterobacteriaceae* and *Veillonellaceae* and a lower abundance of *Blautia*, *Ruminococcaceae* and *Lachnospiraceae* is present in cirrhotic patients [158]. Here we see that there is a significant alteration of the bile acid pool in NALFD as represented by a marked reduction in secondary BAs relative to primary BAs and is accompanied by a dysbiosis of the gut microbiota. Concomitantly, decreased activation of FXR and TGR5 may follow resulting in the dysregulation of metabolic processes. 

#### 3.3.5. NAFLD in Humans 

In humans, liver fibrosis is the main predictor of NAFLD progression and can be assessed noninvasively using transient hepatic elastography [159,160]. Dysregulation of hepatic lipid metabolism can also be reflected in the FA composition of red blood cells (RBCs) [161,162] which can be used as a noninvasive proxy for lipid metabolism in a diseased liver. The recent findings of Cansancao et al. [163] demonstrate this concept who assessed the fatty acid profile of RBCs derived from 89 patients with a NAFLD diagnosis. Analysis showed that patients with advanced fibrosis had higher percentages of palmitic, stearic, oleic, and total MUFAs than patients without. In addition, the ratio of 18:0/16:0 fatty acid type was significantly reduced in patients with advanced fibrosis than those without as well. Assessment of dietary lipid intake by 24 h recall also showed that all patients reported a MUFA intake below the recommended values while 97% reported an increased consumption of saturated fat; no differences in dietary lipid intake were found between patients with or without fibrosis. Further analysis showed that the percentage of PA and fasting insulin was associated with a significantly greater odds ratio for advanced fibrosis [163]. In all, the percentage of PA in RBCs and a reduction of the 18:0/16:0 ratio may be indicative of more advanced NAFLD in humans.

In confirmation of the potent impact of the gut microbiota on liver inflammation, a recent report by Soderborg et al. demonstrated how the gut microbiota derived from 2-week old infants born to obese mothers increases susceptibility to NAFLD and inflammation using a GF mouse model [164]. Interestingly, GF mice colonized with stool from infants born to obese mothers (Inf-ObMB) showed an increase in acetate, propionate, and butyrate concentrations with the largest fold change in butyrate compared to GF mice colonized with stool from infants born to normal weight mothers (Inf-NWMB). Intestinal permeability and bacterial translocation to the liver was also enhanced in these mice as determined by oral gavage of (FITC)-dextran and by total 16s DNA quantification. Analysis of bile acid composition and metabolism showed a loss of fecal BAs with an upregulation of liver bile acid synthesis in Inf-ObMB mice. Hepatic inflammation increased as well, as indicated by an increase in the total macrophage pool and in macrophage mRNA of inflammatory cytokines. To gain further insight monocyte differentiation and eventual macrophage recruitment, the authors also examined the gene expression of proinflammatory cytokines in bone marrow-derived macrophages (BMDMs) isolated from hindlimbs when exposed to LPS for 20 hrs. Though there was an overall reduction of IL-1β, IL-6, TNF and IL-10 expression in BMDMs from Inf-ObMB mice following LPS exposure, their ability to phagocytize *Listeria* was compromised. Lastly, a short-term feeding intervention of 6 weeks with a Western-style diet (WSD) was shown to exacerbate hepatic inflammation as indicated by an upregulation of IL-1β and TNF gene expression in recruited hepatic macrophages as well as a suppression of IL-10 gene expression [164] despite no differences in total hepatic macrophage numbers between both groups. Furthermore, the Pediatric NAFLD Histological Score nearly doubled relative to Inf-NWMB mice given the WSD. In contrast to the above findings of Ye et al., these results posit that the observed increase in butyrate production by infants born to obese mothers may dampen BMDM responsiveness to LPS as SCFAs are known to affect myeloid cell progenitors [165]. This discrepancy may be due to the fact that the observed increase in total SCFA content in Inf-ObMB mice reflect microbial shifts that perhaps enrich butyrate producers to a degree that disrupts an ecological balance as opposed to an exogenous source of butyrate. Still however, it is clear that changes in the early gut microbiome composition in infants born to obese mothers lead to increased gut permeability, reduced macrophage phagocytic activity and bacterial translocation to the liver which contribute to hepatic inflammation and NAFLD in humanized GF mice [164]. Lastly, a WSD can exacerbate hepatic inflammation and accelerate NAFLD onset.

### 3.4. LPS as Immunosuppressive 

Since its first discovery by Cani et al. [14] and as demonstrated in the above studies, LPS has long been considered the most potent activator of innate immunity. To date however, the complex mechanisms by which the host tolerates such a large microbial load and how the gut microbiota itself facilitates this tolerance remain ill-defined. What is most compelling is emergent evidence that demonstrates contrasting immunogenicity of LPS isoforms [166]. For example, early research of lipid A structure and function has indicated that the number of acyl chains is a strong determinant of immune activation [167,168,169] and that penta- and tetra-acylated lipid A structures reduce TLR4 induction [170]. Thus, the microbes from which LPS is derived may determine lipid A’s structure and subsequent immunostimulatory effects. For instance, LPS derived from *Bacteroides* species and *Prevotella copri* of the Bacteroidetes phylum demonstrate a severely impaired compacity to elicit inflammatory responses [166]. To examine the broader impact of LPS isoform variation on gut health and disease, recent advances by d’Hennezel et al. [171] shed new insight on how the gut microbiota facilitates immune tolerance through LPS-mediated antagonization of the TLR4 pathway in healthy humans. Stool samples were collected from nine healthy adults and analyzed by metagenomic whole-genome sequencing (WGS). LPS was purified from each donor and the immunostimulatory potency of each fecal LPS was assessed by stimulating human primary peripheral blood mononuclear cells (PBMCs) where IL-10, IL-6, IL-8, TNF-α, IL-12p70 and IL-1β were measured in parallel. It was found that all LPS samples showed ≥2 magnitude lower stimulatory potency compared to *E. coli* LPS, in which IL-6 and IL-1β were shown as representative results, and many were immunologically silent regardless of dosage [171]; these results were confirmed when stimulating human hTLR2 and hTLR4—NF-κB reporter cell lines. When PBMCs were cotreated with fecal LPS prior to stimulation with *E. coli* LPS, fecal LPS inhibited IL-6 and IL-β production elicited by exposure to *E. coli* LPS. 

To determine the microbial source of the derived LPS, metagenomic WGS was performed on fecal samples from each volunteer. Analysis revealed that distribution of the main phyla, as determined by using MetaPhlAn2 [172], was similar across each volunteer and not distinguishable from 140 healthy-donor samples in the HMP1 suggesting that these samples were representative of the general population [171]. Both sample cohorts were then analyzed using the HUMAnN2 algorithm to determine inferred gene ontology (GO) functions related to LPS biosynthesis. Interestingly, species of the phylum Bacteroidetes dominated the three main GO terms related to LPS biosynthesis (GO:0009245: lipid A biosynthetic process; GO:0009244: LPS core region biosynthetic process; GO:0009103: LPS biosynthetic process). Specifically, Bacteroidetes contributed 79% of the LPS biosynthesis in the healthy volunteers whereas a 92.4% contribution was observed in HMP1 samples. Additional analysis revealed that *Bacteroides ovatus*, *B. uniforms* and *B. vulgatus*, members of the Bacteroidales order, dominated LPS production in both cohorts. The immunosuppressive effects of Bacteroidales species (*Bacteroides, Prevotella* and *Alistipes* spp.) were then examined in PBMCs and showed that all tested members of the Bacteroidales order (*Alistipes putredinis*, *A. finegoldii*, *A. shahii*, *Bacteroides dorei*, *Prevotella copri*) produced a LPS of low immunostimulatory capacity and induced a potent reduction in TNF-α and IL-6 production relative to *E. coli*. [171]. Structural analysis of the lipid A domain from the studied members of the Bacteroidales order by matrix-assisted laser desorption ionization—time of flight mass spectrometry also indicated that immunosilent and immunoinhibitory underacylated lipid A structures were conserved across all tested microbes. Lastly, extracellular polymeric substances (EPS), which are secreted cell-associated matrices that contain biologically active molecules [173], were screened. EPSs derived mainly from the Bacteroidales order suppressed LPS-TLR4 derived cytokine production in human immune cells 4- to 20-fold times despite exhibiting high LPS content [171]. Taken together, these results show that total LPS produced in healthy adult humans is immunoinhibitory and the source of this LPS type is primary derived from species of the Bacteroidales order. These findings challenge the prevailing few that posit LPS as the most immunostimulatory metabolite derived from the gut microbiota. Instead, microbe of origin and subsequent LPS isoforms play a larger role in determining the immunogenicity of microbial communities.

## 4. Endocannabinoid System and the Microbiota-Emerging Area

Recently, the obese state has been associated with an increased endocannabinoid (ECS) system tone and enhancing host energy regulation [174]. The ECS is comprised of cannabinoid (CB) receptor 1 (CB1 [175]), CB2 [176] and the endogenous ligands for CB1 and CB2 derived from membrane bound arachidonic acid, *N*-arachidonoylethanolamine (anandamide, AEA [177]) and 2-arachidonoylglycerol (2-AG [178]). AEA is produced from *N*-arachidonoylphosphatidylethanolamine (NAPE) by hydrolysis with *N*-acylphosphatidylethanolamine-hyrdrolyzing phospholipase D (NAPE-PLD) [179]. Its degradation is primarily from fatty acid amide hydrolase (FAAH) [180]. 2-AG is synthesized from hydrolysis of diacylglycerol sn-1-selective diacylglycerol lipases (DAGL)-α and degraded by monoacylglycerol lipase (MAGL) [179]. Furthermore, AEA can be oxidized by cyclooxygenase-2 (COX-2) to produce prostaglandin glyceryl esters, which have been shown to regulate inflammation [179]. Several groups have shown that CB1 and CB2 are widely distributed throughout the body and both genetic and pharmacologic impairments have shown to protect against the development of obesity, steatosis and inflammation [179,181,182]. In a study comparing several animal models of obesity, Muccioli et al. showed the ECS activity in the colon and adipose tissue was controlled by the gut microbiota [181]. 

The ECS, together with the gut microbiota is involved with gut barrier homeostasis, where ECS regulates gastrointestinal motility [179]. Recently evidence supports the notion that ECS is linked to IBD pathogenesis [176], obesity [182] and diabetes [183]. Several groups have shown AEA is increased in the obese state in the colon and in subcutaneous adipose tissue, where high-fat feeding increased NAPE-PLD and decreased FAAH mRNA expression [181,182]. Sharkey and Wiley [179] describe that inhibition of FAAH to block AEA blocked the development of colitis mediating by blocking of CB1 and CB2. Interestingly, some strains of microbes may minimize visceral sensitivity by upregulating CB2 expression in the intestinal epithelium of rats [184], but conversely in humans there was a reduction in CB2 expression [185]. Muccioli et al. in 2010 show LPS is a potent stimulator of ECS synthesis [181]. In 2012, Cani et al. [186] showed prebiotic treatment drastically affected the gut microbiota and subsequent metabolic endotoxemia by promoting the production of GLP-1 and glucagon-like peptide 2 (GLP-2) by increasing the differentiation of stem cells into enteroendocrine L-cells. Geurts et al. [187] found enhanced inflammatory tone, with increase in IL-1, F4/80, MCP-1 CD11c and CD68 mRNA expression in adipose tissue in genetically type-2 diabetic mice (*db/db*), linking ECS and low-grade inflammatory tone, which regulated apelin and its receptor APJ expression. What is unknown are the mechanisms in how the microbiota participates in ECS and obesity, to control gut permeability and subsequent metabolic endotoxemia. Furthermore, the role of individual microbes and/or microbial metabolites are currently being investigated to understand the mechanisms the contribution to host–microbe mutualism. 

## 5. Conclusions

The present review highlights the role of dietary lipid consumption and concurrent changes in the gut microbiota as well as their metabolites in modulating host inflammatory responses. Diets high in SFAs remain a potent risk factor for inflammatory-based disorders but supplementation with omega-3 fatty acids and dietary fiber may prove to attenuate inflammation. Lastly, an increase in endocannabinoid system tone is associated with obesity but the extent to which it is involved in gut barrier homeostasis remains ill-defined. Collectively, we and others [1,26] support the need for improved spatial and temporal investigations of the gut microbiota, particularly at the species and strain level, to elucidate the causative role of the gut microbiota in host inflammatory-based diseases.
